# Single-dose replicon particle vaccine provides complete protection against Crimean-Congo hemorrhagic fever virus in mice

**DOI:** 10.1080/22221751.2019.1601030

**Published:** 2019-04-04

**Authors:** Florine E. M. Scholte, Jessica R. Spengler, Stephen R. Welch, Jessica R. Harmon, JoAnn D. Coleman-McCray, Brendan T. Freitas, Markus H. Kainulainen, Scott D. Pegan, Stuart T. Nichol, Éric Bergeron, Christina F. Spiropoulou

**Affiliations:** a Viral Special Pathogens Branch, Division of High-Consequence Pathogens and Pathology, National Center for Emerging and Zoonotic Infectious Diseases, Centers for Disease Control and Prevention, Atlanta, USA; b Department of Pharmaceutical and Biomedical Sciences, University of Georgia, Athens, USA

Crimean-Congo hemorrhagic fever virus (CCHFV) is an emerging tick-borne virus from the family *Nairoviridae* that frequently causes lethal disease in humans. CCHFV has a wide geographic distribution, and cases have been reported in Africa, Asia, the Middle East, and Europe. Recent autochthonous cases in Spain demonstrate the emergence of CCHFV in previously naïve regions [1]. CCHFV is considered a public health threat due to its epidemic potential, high case fatality rate, and lack of treatment options. Currently, no antivirals or licensed vaccines with proven efficacy are available. CCHFV was recently included on the World Health Organization Research and Development Blueprint list of infectious agents critically needing effective prophylaxis and therapeutics to prevent major outbreaks. Development of a safe and efficacious vaccine is critical for restricting future outbreaks and preventing disease in endemic countries. In an outbreak situation, a CCHFV vaccine should ideally confer maximal protection after administration of a single dose to rapidly protect at-risk populations.

The genome of CCHFV comprises 3 negative-sense single-stranded RNA segments termed small (S), medium (M), and large (L), respectively encoding the nucleoprotein (NP), the glycoprotein precursor (GPC), and the RNA polymerase (L protein). Currently, the most successful experimental CCHFV vaccines require 1–2 boost immunizations to achieve complete protection, which is not practical in an outbreak situation. These vaccines include a live attenuated modified *Vaccinia* Ankara virus (MVA) expressing GPC, and a DNA vaccine expressing NP and ubiquitin-linked versions of GPC-derived Gn and Gc [2,3]. Protection by the MVA-GPC vaccine appears to require both cell-mediated and humoral arms of the acquired immune response [4]. Indeed, during natural infection, CCHF survivors develop virus-specific IgG and CD8^+^ T-cells that persist for years, potentially conferring long-term immunity to reinfection [5,6].

Here we describe the development and assessment of a CCHF virus-like replicon particle (VRP) vaccine candidate. We hypothesized that immunization with VRPs, that closely mimic the structure and composition of authentic CCHFV, might induce a more robust development of CCHFV immunity than current experimental vaccine options.

The method for producing VRPs is based on the reverse genetics system previously described for CCHFV strain IbAr10200 [7,8]. For VRP production, the plasmid containing the full-length M genome segment (pT7-M) is replaced with a plasmid producing the codon-optimized GPC of the Oman-1998 strain (pCAGGS-GPC-Oman, Figure 1(a)). Absence of the M segment in VRP particles limits their replication to a single cycle. To maximize the probability of generating strong immunity to the VRPs, GPC-Oman was selected because it was previously found to improve the capacity of CCHF virus-like particles to enter specialized antigen-presenting cells like human macrophages [9]. Seed stocks of wild-type VRPs were obtained by transfecting cells with the plasmid set depicted in Figure 1(a) (see Supplemental Material for details). VRPs expressing a green fluorescent protein (ZsGreen) were obtained by replacing the plasmid encoding the full-length S genome segment (pT7-S) with a variant that also encodes ZsGreen [8]. Supernatants containing VRPs were harvested 4–5 days post transfection. To obtain higher titres, VRPs were further amplified for 2–3 days in cells transfected with pCAGGS-GPC-Oman, resulting in stocks containing >5 × 10^5^ TCID_50_/mL of either wild-type or ZsGreen VRPs. All stocks were verified by next-generation sequencing (Illumina). To confirm that VRPs are limited to a single replication cycle in the absence of GPC trans-complementation, we passaged VRP-ZsGreen in cells transfected with pCAGGS-GPC-Oman or an empty vector, and monitored fluorescence (Figure 1(b)). Large foci of ZsGreen-positive cells were observed in GPC-transfected cells, while only single ZsGreen-positive cells were detected in the empty vector control. No increase in the number of ZsGreen-positive cells was seen in the transfection control on days 2 and 3, supporting that GPC trans-complementation is required for VRP spread. Passage of day 3 cell supernatants on fresh cells further confirmed the strict requirement of GPC trans-complementation for the production of progeny VRPs. In parallel the complete CCHFV was passaged, which does not require GPC complementation.

**Figure 1.**
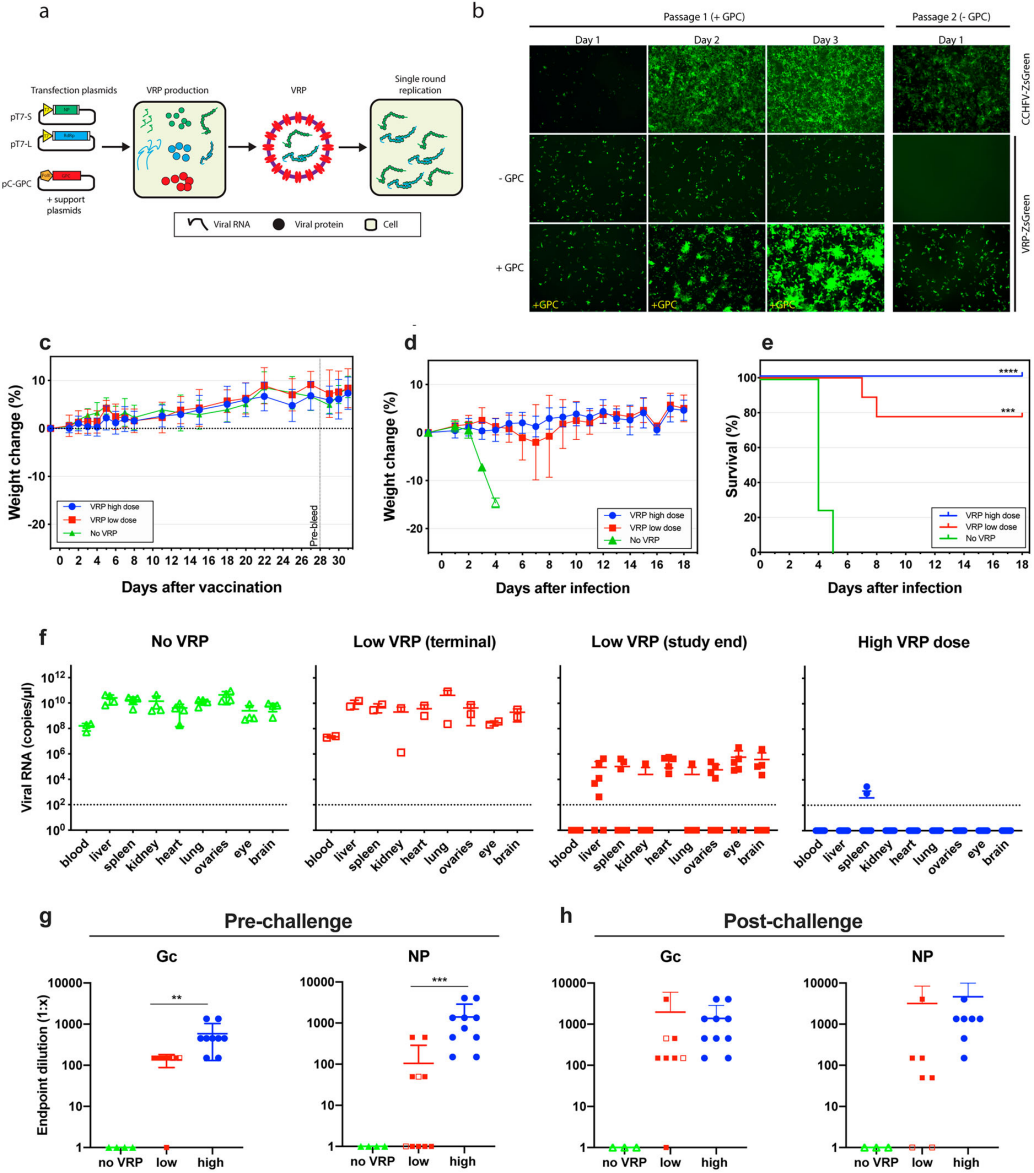
CCHF VRP vaccine protects against lethal challenge in IFNAR^−/−^ mice. (a) Schematic overview of VRP production. Huh7 cells were transfected with plasmids encoding the CCHFV L(pT7-L) and S(pT7-S) genome segment, as well as support plasmids encoding NP, human-codon optimized L, GPC, and T7 RNA polymerase. (b) VRPs are single round particles that require trans-complementation with GPC to propagate. ZsGreen-expressing VRPs were added to Huh7 cells (MOI 0.01) transfected with pCAGGS-GPC-Oman or an empty plasmid. After 3 days, supernatants were transferred to fresh (untransfected) Huh7 cells. ZsG fluorescence was monitored as a measure of VRP replication and spread (10x magnification). Recombinant CCHFV-ZsG (MOI 0.01) was passaged in parallel as a control. (c) Monitoring of mouse weight post vaccination. IFNAR^−/−^ mice were vaccinated with a high dose (10^5^ TCID_50_, *n* = 10), low dose (10^3^ TCID_50_, *n* = 10) or mock-vaccinated (DMEM, *n* = 4). Animals were monitored daily for adverse effects and weight loss. Animal weights are shown as mean ± SD, and represent percentage weight change relative to a baseline set at 1 day before vaccination. Twenty-eight days post vaccination blood was collected via the mandibular vein (shown as the “pre-bleed” vertical line). (d, e) Animals were challenged with a lethal CCHFV dose (100 TCID_50_) 32 days after vaccination. Mouse weight (d) and survival (e) were monitored daily after CCHFV challenge. Animal weights are shown as mean weights ± SD, and represent percentage weight change relative to a baseline set at 1 day before challenge. Statistical significance was determined using a Log-rank (Mantel-Cox) test using GraphPad Prism 8. ***p < 0.001, ****p < 0.0001. (f) The levels of viral RNA was analyzed using quantitative RT-PCR. RNA was isolated from blood and various homogenized tissues either when animals reached clinical end points (“terminal” samples, open symbols) or at the end of the study (“end of study,” closed symbols). (g, h) Levels of IgG antibodies against CCHFV NP and Gc were determined 28 days after vaccination (pre-challenge) (g), and at the end of the study or at time of euthanasia (post-challenge) (h). Open symbols represent animals that succumbed to disease. Closed symbols represent animals that reached study end. One untreated (no VRP) animal could not be sampled post challenge. Statistical significance was determined using Mann-Whitney tests using GraphPad Prism 8, **p < 0.01, ***p < 0.001.

Next, we examined whether single-dose immunization with wild-type VRPs protects against lethal CCHFV challenge in mice lacking type I interferon receptor (IFNAR^−/−^). Eight-week-old female mice were vaccinated subcutaneously with either a high (10^5^ TCID_50_) or a low dose (10^3^ TCID_50_) of VRPs. Control mice were inoculated with Dulbecco’s Modified Eagle Media (DMEM) only. No weight loss or clinical signs were observed in mice in any of the 3 experimental groups during the 32-day post vaccination period (Figure 1(c)). At 28 days post vaccination, blood samples were collected to determine titres of IgG antibodies against NP and Gc. Animals vaccinated with the high VRP dose developed robust levels of IgG antibodies against both NP and Gc (Fig. 1g and Supplementary Material, Table 1). Mice vaccinated with the low dose demonstrated a less robust IgG response overall; only 5/10 of the mice developed anti-NP antibodies, and 9/10 mice developed anti-Gc antibodies.

Thirty-two days post vaccination, animals were subcutaneously challenged with a uniformly lethal dose (100 TCID_50_) of recombinant CCHFV-IbAr10200 (Figure 1(d) and (e)). One mouse was removed from the low dose group prior to challenge due to unrelated health issues (dermatitis). Animals were monitored daily for signs of clinical illness. All mock-vaccinated mice succumbed to infection 4–5 dpi, exhibiting clinical signs including weight loss, hunched posture, ruffled coat, weakness, decreased activity and/or moribundity. Two of the nine mice that received the low dose reached euthanasia criteria at 7 and 8 dpi, exhibiting similar clinical signs to the mock-vaccinated control group. An additional animal in the low dose group experienced 2 consecutive days of weight loss (7–8 dpi), but subsequently recovered and remained healthy for the rest of the study (Supplemental Material, Figure S1). In contrast, none of the mice vaccinated with the high VRP dose exhibited any clinical signs of infection.

To determine if protected animals had detectable levels of CCHFV S RNA at the end of the study, total RNA was isolated from blood and homogenized tissues as previously described [10]. High levels of S RNA were detected in all analyzed tissues of the mock-vaccinated mice and the 2 mice from the low dose group that succumbed to infection (Figure 1(f)). Lower levels of S RNA could be detected in all examined samples of the remaining mice in the low dose group. In contrast, no S RNA could be detected in mice vaccinated with the high dose, except low levels in the spleen of 2 of the 10 animals. Since CCHFV S RNA is also expressed by the VRP vaccine, a virus-specific M segment qRT-PCR was performed to unequivocally discriminate VRP gene expression from CCHFV infection (Supplemental Material, Figure S3). Detection of virus-specific M RNA was limited to animals that succumbed to CCHFV challenge (i.e. all mock-vaccinated controls and 2 mice from the low dose vaccinated group). This supports the absence of CCHFV in protected animals at the study end, and suggests that the high VRP dose results in increased clearance compared to the low dose, potentially conferring sterilizing immunity in some individuals.

Analysis of antibody titres after CCHFV challenge revealed the absence of detectable IgG in mock-vaccinated mice (Figure 1(g) and (h)). The 2 low dose vaccinated mice that succumbed to infection had no detectable antibodies against NP, though antibodies against Gc, using an in-house ELISA, could be detected (Figure 1(g) and (h)). In contrast, all vaccinated animals that survived the challenge (17/19) developed antibodies against NP, and all but one animal (18/19) developed antibodies against Gc. CCHFV challenge strongly boosted anti-NP and Gc antibody titres in mice vaccinated with the low dose. In contrast, the titres of both NP and Gc antibodies only modestly increased in mice that received the high VRP dose, possibly reflecting the absence or lower levels of CCHFV replication than in the low dose group. Further investigation into antibody functions and T-cell responses will be necessary to establish their role in protection.

Altogether, these data confirm the safety and efficacy of a single dose of this novel vaccine in immunocompromised IFNAR^−/−^ mice, an extremely sensitive CCHFV model. This reverse genetics system could easily be modified to potentially further increase its efficacy at a lower dose or to use it as a platform for developing new CCHFV vaccines. For example, we demonstrated that ZsGreen can be inserted into the S segment. Using this strategy, we can modify the platform to elicit immunity toward other antigens, potentially generating a bivalent vaccine. In addition, the immunogenicity and efficacy at lower doses could be further optimized by modifying the NP and L proteins. For instance, the deubiquitinase activity of the ovarian tumour domain of the L protein could be modified to promote the production of type-I interferons [11], which are important for optimal antigen presentation and subsequent development of memory and effector T cells [12]. The immune response associated with CCHF survival or experimental vaccines is unknown. VRPs recapitulate most aspects of CCHFV replication without causing disease, therefore they could be a useful model to define the protective immune responses without the biosafety concerns associated with live CCHFV experimentation. In conclusion, these data support CCHF VRPs as a promising vaccine platform that could help reduce the public health threat of CCHFV. Future studies should focus on further characterization of the immune response to vaccination, efficacy of timing of vaccination (i.e. pre- and post-exposure), and elucidating the mechanisms of protection.

## Supplementary Material

Supplemental Material
