# Supplementary material for: Single-dose replicon particle vaccine provides complete protection against Crimean-Congo hemorrhagic fever virus in mice
Source: Emerg Microbes Infect. 2019 Apr 4;8(1):575–8. doi: 10.1080/22221751.2019.1601030 (PMC6455139; doi:10.1080/22221751.2019.1601030)
Supplement: Supplemental Material [file TEMI_A_1601030_SM3481.docx]

**Supplementary Material**

**VRP production**

Six-well plates were seeded with 3.5 × 10^5^ Huh7 cells/well 1 day prior to transfection in 3 mL of DMEM supplemented with 1% non-essential amino acids, 1 mM sodium pyruvate, and 10% FBS. 16–24 h later, cells were transfected with pT7-S (1 μg) or pT7-S-P2A-zsGreen (1 μg) to produce fluorescent reporter protein^8^, together with pT7-L (1 μg), pCAGGS-L (0.33 μg), pCAGGS-N (0.66 μg), pCAGGS-GPC-Oman (1 μg), and pCAGGS-T7 (1 μg), combined with 12.5 μL of Mirus LT1 transfection reagent (Mirus Bio, Madison, WI, USA) in 250 μL of OPTI-MEM (Life Technologies, Grand Island, NY, USA) (Fig. 1a). Supernatants containing VRPs were harvested 4–5 days post transfection. VRP stocks were titrated by 50 percent tissue culture infective dose (TCID_50_) on BSR/T7 cells using the Reed-Muench method. Positive wells were scored based on the detection of at least one CCHFV NP positive cells detectable by immunofluorescence using a rabbit anti-NP antibody (#04-0011, IBT Bioservices) and Alexa-488 goat anti-rabbit secondary antibody.

**Animal experiments**

Animal work was approved by the Centers for Disease Control and Prevention IACUC and conducted in accordance with the *Guide for the Care and Use of Laboratory Animals, 8^th^ Edition*, at an AAALAC-accredited facility. Female B6.129S2-*Ifnar1^tm1Agt^*/Mmjax mice (MMRRC 032045-JAX; Jackson Laboratories), 8 weeks old and 17 – 21g at vaccination, were housed in a climate-controlled laboratory with a 12 h day/12 h night cycle. All animals were given sterile water and food *ad libitum*, and group housed (4 – 5/cage) on autoclaved corn cob bedding (Bed-o'Cobs® ¼”, Anderson Lab Bedding), in an isolator-caging system (Thoren Caging, Inc., Hazleton, PA, USA) with a HEPA-filtered inlet and exhaust air supply. The cage environment was enriched with shredded paper, cotton nestlets, and a hide-away structure. Mice were vaccinated subcutaneously (SC) under isoflurane anesthesia with either a high VRP dose (target dose: 1x10^5^, actual dose: 4.39x10^5^) or a low dose (target dose: 1x10^3^, actual dose: 4.64x10^3^). Post vaccination (pre-challenge) blood collection was performed via the submandibular vein using a lancet (Monolet^TM^). Thirty-two days post vaccination, animals were challenged SC under isoflurane anesthesia with a uniformly lethal dose (target dose: 100 TCID_50_, actual dose: 37 TCID_50_) of recombinant CCHFV-IbAr10200 (GenBank ﻿KJ648914, ﻿KJ648915, and ﻿KJ648913). Clinical signs were scored based on 14 parameters: 2 points each for QDR (quiet, dull, responsive) disposition, hunched back, or ruffled coat; 3 points each for dehydration or abnormal huddling/hypoactivity; 5 points each for presence of neurological signs (ataxia, circling, tremors, or paresis), abnormal breathing, or anemia; 7 points for weight loss of >20% from baseline (d-1); 10 points each for inability to bear weight, paralysis, frank hemorrhage/bleeding, moribund state, or weight loss of >25% from baseline. Animals were humanely euthanized when end-point criteria were reached (clinical score ≥ 10), or at study completion (18 days post challenge).

**CCHFV-Gc Construct Design**

The construct of the ectodomain of the CCHFV Glycoprotein (Gc) was synthesized by cloning the Oman M segment coding region corresponding to the N-terminal signal peptide (aa. 1-29) followed by residues (999-1598) (ALT31693.1) and a C-terminal HisTag into a pcDNA3.1(+) vector.

**CCHFV-Gc Expression and Purification**

The Gc ectodomain expression plasmid was transformed into HEK Expi293 cells. Cells were grown in 300mL cultures at 37°C in Expi293 Expression Medium (Gibco A14351) for 72 hr until culture viability dropped below 50%. Cells were harvested by centrifugation at 2500*g* for 30 min and stored at -80°C. Frozen cell pellets were thawed at room temperature for 30 min and treated with 30mL M-PER (Thermo 78501) for 10 min with shaking. Cell lysates were separated into soluble and insoluble fractions via centrifugation at 14,000*g* for 15 min. The soluble fractions were syringe-filtered through 0.22 μm membranes. Filtered samples were loaded over a charged 5mL HisTrap HP column, washed with wash buffer [20 mM NaPO_4_ (pH 7.6), 500 mM NaCl, and 20 mM Imidizole] until UV baseline, and eluted over a 3CV gradient from 0-100% elution buffer [20 mM NaPO_4_ (pH 7.6), 500 mM NaCl, and 500 mM Imidizole] collecting 2 mL fractions. Pure fractions were pooled and samples were dialyzed twice in 5L of PBS for 2 hr at room temperature using the 3.5K Pur-A-Lyzer dialysis kit (Sigma PURG35020).

**ELISA**

To determine serum neutralizing antibody titers, plasma was separated from whole blood collected in lithium heparin tubes by centrifuging 3 min at 8000 rpm. Samples were inactivated using gamma irradiation (5 million rads from a ^60^Co source). CCHFV NP IgG was detected using a commercial ELISA kit (Alpha Diagnostics International AE-320400-1); CCHFV Gc IgG levels were determined using an in-house ELISA assay using purified Gc ectodomain bound to nickel-coated 96-well plates (1 µg per well).
